# A differentiable simulation package for performing inference of synchrotron-radiation-based diagnostics

**DOI:** 10.1107/S1600577524000663

**Published:** 2024-02-16

**Authors:** Robbie Watt, Brendan O’Shea

**Affiliations:** a SLAC National Accelerator Laboratory, 2575 Sand Hill Road, Menlo Park, CA, USA; Australian Synchrotron, Australia

**Keywords:** accelerator diagnostics, differentiable simulations, machine learning

## Abstract

The direction of particle accelerator development is ever-increasing beam quality, currents and repetition rates. This poses a challenge to traditional diagnostics that directly intercept the beam due to the mutual destruction of both the beam and the diagnostic. An alternative approach is to infer beam parameters non-invasively from the synchrotron radiation emitted in bending magnets. However, inferring the beam distribution from a measured radiation pattern is a complex and computationally expensive task. To address this challenge we present *SYRIPY* (*SYnchrotron Radiation In PYthon*), a software package intended as a tool for performing inference of synchrotron-radiation-based diagnostics. *SYRIPY* has been developed using *PyTorch*, which makes it both differentiable and able to leverage the high performance of GPUs, two vital characteristics for performing statistical inference. The package consists of three modules: a particle tracker, Lienard–Wiechert solver and Fourier optics propagator, allowing start-to-end simulation of synchrotron radiation detection to be carried out. *SYRIPY* has been benchmarked against *SRW*, the prevalent numerical package in the field, showing good agreement and up to a 50× speed improvement. Finally, we have demonstrated how *SYRIPY* can be used to perform Bayesian inference of beam parameters using stochastic variational inference.

## Abstract 2

*SYRIPY* (*SYnchrotron Radiation In PYthon*) is a new package for modelling the production and propagation of synchrotron radiation. The package has been developed using *PyTorch*, making it both differentiable and able to leverage the high performance of GPUs.

## 1. Introduction

The field of accelerator development is continually pushing boundaries to generate increasingly brighter beams, elevate peak currents and enhance repetition rates. These high-intensity beams serve as unique tools for unveiling new insights across a diverse range of research areas, from ultra-high-gradient plasma wakefield accelerators to probing strong field quantum electrodynamics (Yakimenko *et al.*, 2016 ▸). For instance, the Facility for Advanced Accelerator Experimental Tests II (FACET-II) will soon boast the capability of delivering beams with a charge of 2 nC, at an energy of 10 GeV, with a normalized transverse emittance of less than 10 µm, and up to 200 kA in peak current (Yakimenko *et al.*, 2019 ▸). However, these intense beams present a considerable challenge to traditional accelerator diagnostics, particularly those that require placing material in the beam’s path. For example, beam size measurements rely on capturing optical transition radiation emitted as the beam traverses a thin foil. These foils are subject to quick deterioration due to surface heating (Stupakov, 2013 ▸), resulting in operational difficulties, including escalating costs and time expenditure. Moreover, these destructive diagnostics negatively impact the downstream beam quality, thus barring their simultaneous operation with an experiment. As such, there is a growing preference for single-shot, non-invasive diagnostics that leverage machine learning to overcome these hurdles (Emma *et al.*, 2018 ▸, 2021 ▸).

As a beam travels along an accelerator, it passes through bending magnets and emits synchrotron radiation. This radiation is common to both linear and circular accelerators, making it a promising candidate for a single-shot, non-invasive diagnostic. Upon entering or exiting a bending magnet, the beam is subject to rapidly fluctuating fringe fields. In such conditions, the intensity of the radiation produced can eclipse that of standard synchrotron radiation, a phenomenon known as edge radiation (Titov & Yarov, 1991 ▸; Chubar, 1995*a* ▸; Bosch, 1999 ▸; Geloni *et al.*, 2009 ▸). When the beam traverses two successive bending magnets, the emitted radiation can interfere, resulting in a ringing intensity profile. These rings exhibit a high sensitivity to the beam’s size and divergence, which makes edge radiation a prime candidate for a single-shot, non-invasive diagnostic. This potential has been previously explored in applications at both the Siberia-1 electron storage ring and the FERMI free-electron laser (Chubar, 1995*b* ▸; Fiorito *et al.*, 2014 ▸).

To extract beam information from a measured intensity profile through statistical inference, a model of the system is required. This cannot be achieved analytically, making a numerical simulation necessary. A number of publicly available software packages exist for this purpose, including *SPECTRA* (Tanaka & Kitamura, 2001 ▸) and *Synchrotron Radiation Workshop* (*SRW*) (Chubar & Elleaume, 1998 ▸). These packages are widely used and extensively benchmarked against experimental results. However, when applying statistical inference, a large number of simulations must be carried out. The existing software packages capable of modelling edge radiation are limited to CPU-based operation, which is suboptimal for this objective. Therefore, we have developed a new package *SYRIPY* (*SYnchrotron Radiation In PYthon*) built upon the *PyTorch* library (Paszke *et al.*, 2019 ▸). *SYRIPY* is specifically designed as as a tool for performing inference of synchrotron-radiation-based diagnostics. Through *PyTorch*, the code runs natively on graphics processing units (GPUs), allowing us to make use of the massively parallel architecture for high numerical efficiency. Furthermore, *SYRIPY* can utilize *PyTorch*’s automatic differentiation package to calculate the gradient of output intensity profiles with respect to simulation inputs. This high efficiency and gradient information are invaluable tools for applying inference schemes in high-dimensional spaces.

In this paper we will review and demonstrate our new synchrotron radiation toolkit *SYRIPY*. We will begin in Section 2 by discussing the system of equations which the package solves and detail the specific numerical implementation. In Section 3 we will provide benchmark results, comparing against both analytical and numerical calculations. Finally, we will demonstrate an application of the package, using it to perform Bayesian inference on mock experimental data.

## 2. Theory and numerical implementation

**Figure 1:**
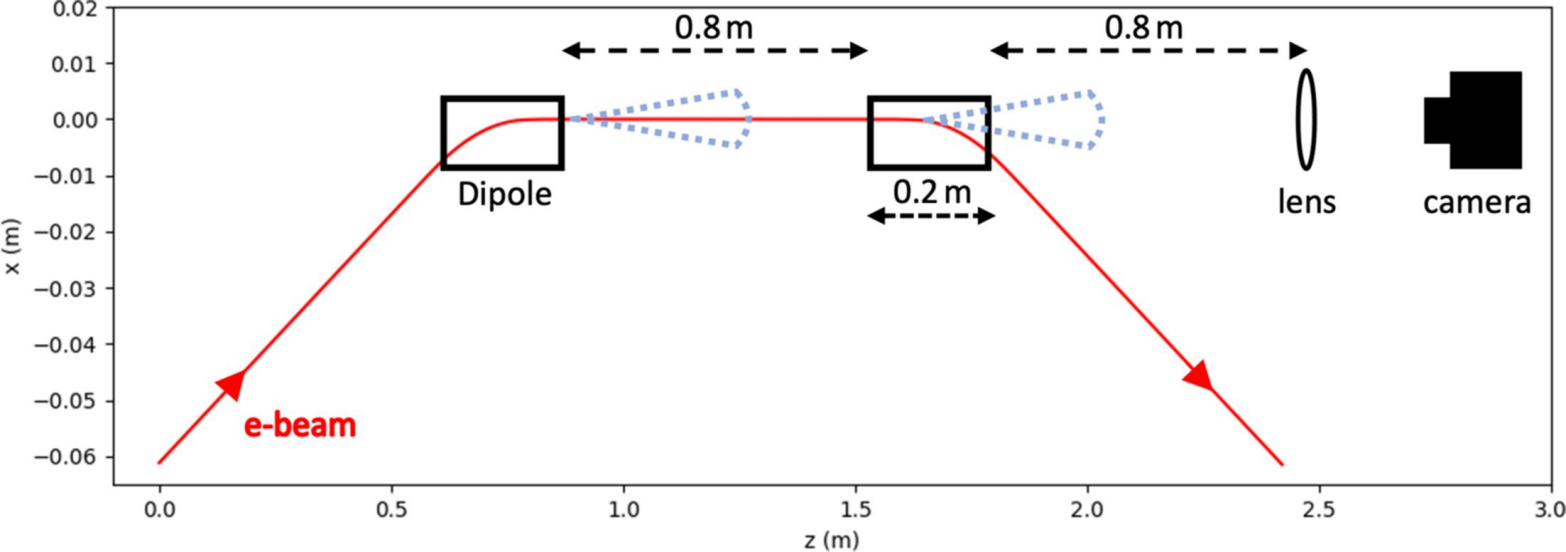
Diagram showing the production and detection of edge-radiation in the central part of a bunch compressor. This shows a 300 MeV electron beam passing through two 0.5 T dipoles and emitting synchrotron radiation. The field is then propagated to the detector at the Fourier plane of a *f* = 10 cm lens.

A start-to-end simulation of synchrotron radiation production and detection, spanning from the initial electron beam parameters to the expected intensity profile at a detector, can be divided into three stages. First, electron trajectories are obtained by tracking the electron beam through the region of interest. Secondly, using these trajectories, the electromagnetic field at an initial downstream wavefront is calculated. Finally, the field is propagated through optical elements to the detector plane. These stages are demonstrated in Fig. 1 ▸, which shows a diagram of the production and detection of edge radiation in the centre of a bunch compressor. In this section we will detail the theory and our numerical implementation used to solve this system. This implementation is highly parallelizable over a number of simulation parameters, including electrons, observation points and time samples. Therefore, parallelized hardware (*i.e.* GPUs) are ideal for carrying out these calculations.

### 2.1. Particle tracking

The first part of the calculation consists of sampling electrons from the beam and generating their trajectory through the region of interest. If we assume the interaction between electrons within the beam is negligible, their motion is governed by the Lorentz equation of motion, 

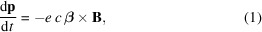

where **p** is the electron momentum, **β** is the relativistic velocity, *c* is the speed of light and *e* the electron charge. **B** ≡ **B**(**r**) denotes the used defined magnetic field, which can consist of drift spaces, dipoles and quadrapoles. Equation (1) is solved using a fourth-order Runge–Kutta scheme. Parallelizing the calculation over multiple electrons within the beam is trivial due to the independence of trajectories.

### 2.2. Radiation solver

Having generated a sample of electron trajectories, the next step is to calculate the resulting synchrotron radiation at a downstream plane (*i.e.* wavefront). This calculation is performed for each sampled trajectory individually. The electromagnetic field due to the arbitrary motion of a single electron is given by the Liénard–Wiechert scalar and vector potentials (Jackson, 1999 ▸; Landau, 2013 ▸), 

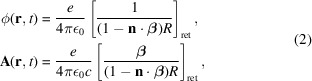

where SI units are used, **r** = (*x*, *y*, *z*) is the observation point, *t* is the observation time, *R* = |**r** − **r**_e_| is the distance between the electron and the observation point, **r**_e_ is the electron position, **n** is the unit vector pointing from electron to the observation point (*i.e.* **n** = **R**/|**R**|), ε_0_ is the electric constant and […]_ret_ denotes that the term inside the brackets is calculated at the retarded time 

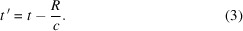

The more familiar electric field can be expressed in terms of the scalar and vector potentials through the following definition,

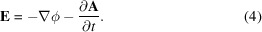

Most diagnostics are only sensitive to radiation over a limited spectral range. Therefore, it is more efficient to calculate the electric field in the frequency domain as opposed to the temporal domain. Applying a Fourier transform to equation (4) yields 

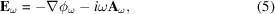

where 

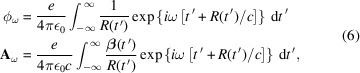

and ω is the angular frequency of the field. Using equations (5) and (6), the Fourier domain electric field is given by (Chubar, 1995*a* ▸) 

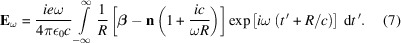

By calculating equation (7) over a grid of observation points the initial wavefront is obtained. Each of these observation points is independent, which once again enables the calculation to be easily parallelized.

Equation (7) is of the form 

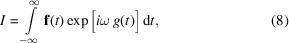

where **f**(*t*) is a slowly varying function and exp[*i*ω*g*(*t*)] oscillates rapidly. This makes solving equation (7) numericaly infeasible using standard quadrature methods. To understand why, we can study the setup in Fig. 1 ▸, taking the electron energy and dipole field strength to be ∼100 MeV and ∼0.1 T, respectively. The electrons are moving close to *c*, so will take 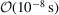 to traverse the setup, which sets the limits of the integration. If we are performing the calculation at the peak of the synchrotron emission spectrum [ω ≃ 10^15^, using equation (24)] the oscillating part of the integral will have a period of ∼10^−15^ s. To prevent large numerical errors, the integrand must be densely sampled such that these oscillations are resolved. For our example this would require at least 10^7^ samples which is unpractical.

Solving equation (7) using practical computational resources requires quadrature methods specifically designed for highly oscillatory functions. To apply these, we start by splitting the integral into three parts, 

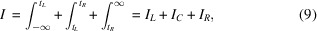

where *I*_*C*_ integrates over the the trajectory in which the electron passes through the region of interest. Outside the region of interest (*I*_*L*/*R*_), we assume the electron travels to infinity with a constant velocity **β**_*L*/*R*_. Ignoring *I*_*L*/*R*_ would result in the emission of spurious radiation from the creation and destruction of the electron at *t*_*L*_ and *t*_*R*_. The electron’s position as a function of time is simply given by **R** = *c* **β**_*L*/*R*_ (*t* − *t*_*L*/*R*_) + **R**_*L*/*R*_, where **R**_*L*/*R*_ is the location of the electron at the integral boundary *t*_*L*/*R*_. Using this, derivatives of **f**(*t*) and *g*(*t*) can be computed to any order, allowing us to solve *I*_*L*/*R*_ using an asymptotic expansion. This involves successively applying integration by parts to equation (8), generating a sequence with terms increasing in order ω^−1^. To first order this gives (Stein & Murphy, 1993 ▸) 

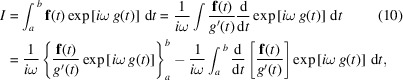

where the first term is an approximation of the integral and the second term is the error. To continue the expansion, the same process is applied to the error. For the systems of interest to this work, ω ≫ 1, making the expansion converge rapidly. Therefore, a first-order expansion is found to be sufficient.

The trajectory between *t*_*L*_ and *t*_*R*_ is a complicated parametric function of the user-defined magnetic field. Information about **f**(*t*), *g*(*t*) and their derivatives at the boundaries is insufficient for solving *I*_*C*_. Therefore, it is not possible to apply an asymptotic expansion and instead we adopt Filon’s method (Filon, 1930 ▸). This method shares similarities with the commonly used Simpson’s rule, as a quadratic approximation is applied to the non-oscillating part of the integral. Before applying the Filon method, we first remove the irregular, non-stationary phase using a change of variables *x* = *g*(*t*), 

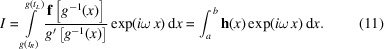

The integral is then discretized into *n* intervals and **h** is interpolated by a quadratic at the ends and centre points (*x*_1_, *x*_2_ and *x*_3_) of each interval, *i.e.* **h**(*x*) ≃ **v**(*x*) = **c**_1_ + **c**_2_ *x* + **c**_3_ *x* ^2^. The parameters of the quadratic fit **c**_*i*_ within each interval are obtained by solving the linear system 

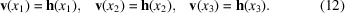

Applying this quadratic approximation to equation (11) yields 

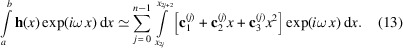

Euler’s formula is used to express the complex exponential in terms of sine and cosine functions, allowing the integral within each interval to be solved using the analytical formula for the moments 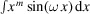 and 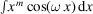 . For a fixed interval size, the error in the approximation of equation (13) decays as 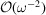 (Stein & Murphy, 1993 ▸). This is the same as the first-order asymptotic expansion used to calculate *I*_*L*/*R*_.

#### 2.2.1. Increasing numerical efficiency

Equations (10) and (13) can be readily solved using double-precision floating point format (FP64) to obtain the initial wavefront field. However, leveraging single-precision (FP32) is advantageous, as GPUs are generally optimized for this format. Nvidia GPUs based on the Ampere architecture (for example, the Nvidia RTX A6000 used to perform simulations for this work) have an FP32 to FP64 theoretical peak performance ratio of 32:1. We cannot directly perform the calculation using FP32 as numerical errors are likely to arise from catastrophic cancellation. To understand why, we can study the dominant emission region of equation (11). This occurs when the denominator *g*′(*t*) = 1 − **n** · **β** is small. Given that the electron is highly relativistic, **n** · **β** ≃ 1 when the electron is moving towards the observer. Obtaining *g*′(*t*) requires taking the difference between two similar numbers, resulting in a large relative error if FP32 is used. To avoid this cancellation error, we can apply a small observation angle approximation (*i.e.* *Z* = |*z* − *z*_e_| ≫ *X* = |*x* − *x*_e_|, *Y* = |*y* − *y*_e_| and β_*z*_ ≫ β_*x*_, β_*y*_) which allows us to rewrite the phase gradient *g*′(*t*) and phase *g*(*t*) as 

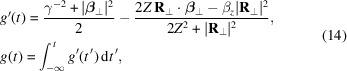

where γ is the Lorentz factor and **R**_⊥_ and **β**_⊥_ are the transverse relative position and velocity, respectively. Defining the phase gradient with equation (14) avoids taking the difference between similar numbers.

**Figure 2:**
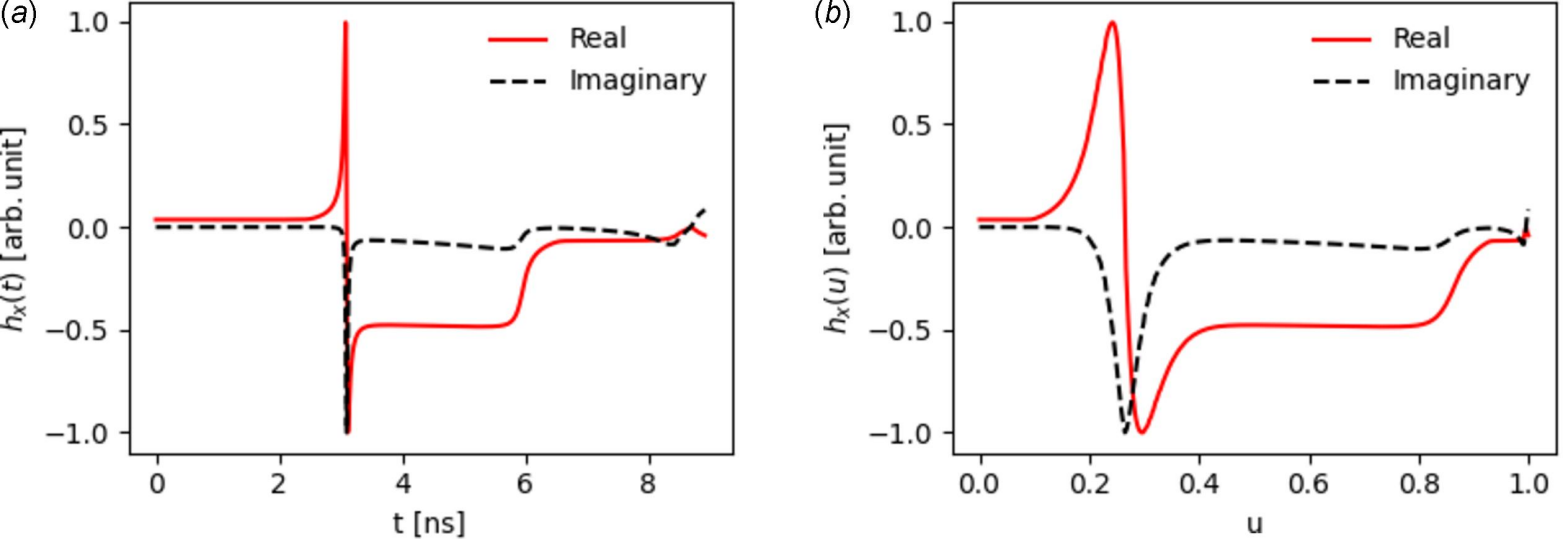
Real and imaginary parts of *h*_*x*_(*x*) with (*a*) showing the function plotted against evenly spaced time samples and (*b*) showing the function plotted against *u*.

Realizing that the radiation emission is dominated in the region where *g*′(*t*) is small allows us to make further numerical improvements. To obtain small numerical errors from Filon’s method, **h**(*x*) must be accurately approximated by a quadratic within each interval. Fig. 2 ▸(*a*) shows a plot of *h*_*x*_(*t*) [the *x*-component of **h**(*x*)] for the system shown in Fig. 1 ▸. As the electron passes through the compressor, the integrand experiences both sharp peaks and flat regions. To approximate the flat regions with a quadratic, a low density of samples is required, whereas the sharp peaks require a high density of samples. Therefore, solving equation (13) using evenly spaced time samples is inefficient.

A more efficient approach would redistribute the samples such that the density is higher when **h**(*x*) changes rapidly, *i.e.* when |[*g*′(*t*)^−1^]′| is large. This can be achieved using inverse transform sampling. Here, the cumulative distribution function (CDF) of a target distribution *p*(*t*) ∝ |[*g*′(*t*)^−1^]′| is calculated, 

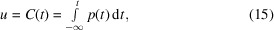

and its inverse *t* = *C* ^−1^(*u*) obtained. If we take a set of evenly spaced samples in *u* and transform them according to *C* ^−1^(*u*), we will obtain irregular spaced samples in *t* with the required density. This is demonstrated in Fig. 2 ▸(*b*) showing the same function as (*a*) now plotted against *u*. The peaks are now wider, meaning **h**(*x*) can be accurately approximated by a quadratic using fewer samples.

So far, we have only redistributed the samples according to the emission observed at a single point. However, for an observation point at a different location along the *x*-dimension (the transverse dimension in which the beam is bent) the target distribution will change. As **n** and **β** are now parallel at a different part of the trajectory, the peaks in Fig. 2 ▸(*a*) will shift. The updated time samples will not be optimal for this new observation point. To solve this issue, |[*g*_*i*_′(*t*′)^−1^]′| is calculated at a set of *M* locations along *x*, and the new target distribution is given by the maximum over this set at any given value of *t*, 

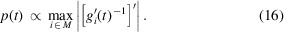

**Figure 3:**
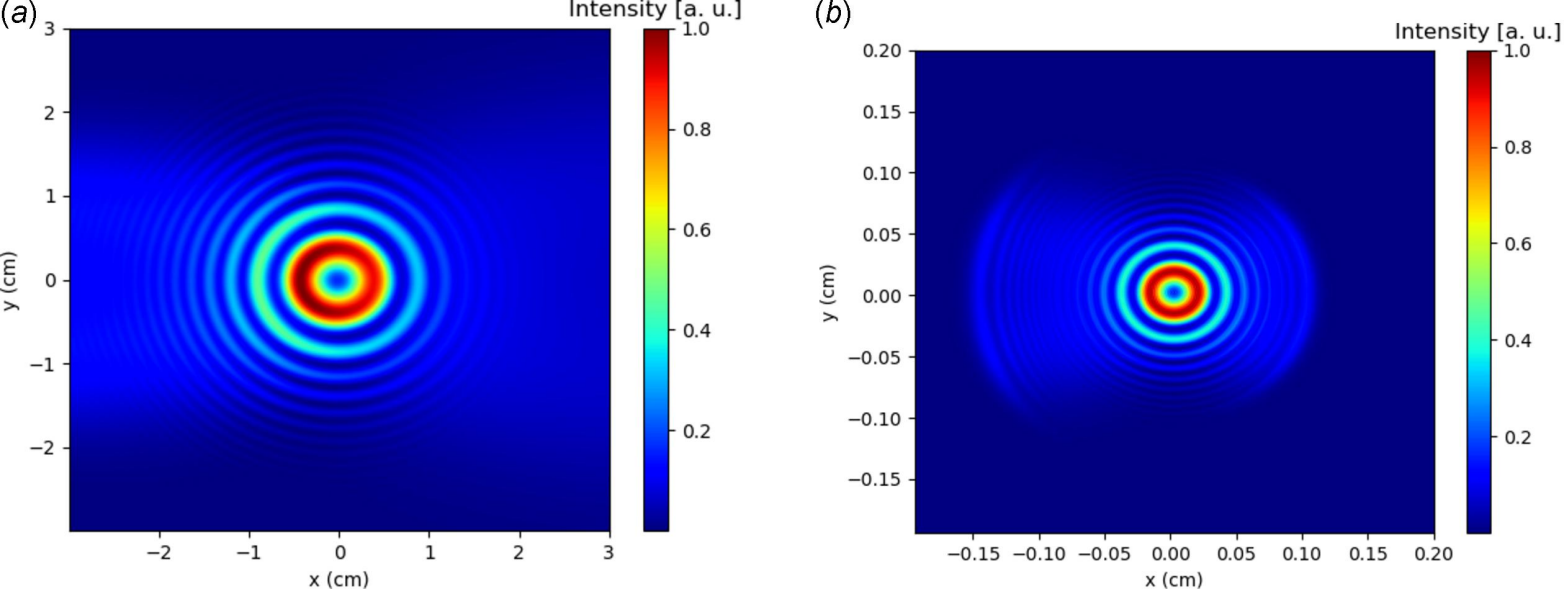
Wavefront intensity profiles at (*a*) the initial wavefront and (*b*) the detector plane for the setup shown in Fig. 1 ▸. The radiation wavelength is λ = 5 µm and total samples *N*_*x*_ *N*_*y*_ = 500^2^.

For a single observation point, redistributing the samples requires additional overheads, increasing the time taken to perform the calculation. However, this process is only carried out over a limited number of observation points, much less than the total number in the 2D wavefront (*i.e.* 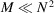 where *N* is the number of wavefront observation points in both *x* and *y*). Therefore, the overhead time is small compared with the total simulation time. On top of this, as we will see in the next section, to calculate the intensity from a beam with finite emittance involves summing over multiple electron trajectories. Given that the beam is small, the emission peaks will occur at similar locations for all trajectories. Therefore, this process only needs to be carried out for the central trajectory, and the updated time samples used for all electrons in the beam. We have found for the example calculation shown in Fig. 3 ▸ that this process can reduce the number of samples by a factor of five without a noticeable increase in the error.

#### 2.2.2. Emission from an electron beam

When measuring the radiation profile with a camera, the quantity that is directly obtained is the total photon flux density from a beam of electrons (number of photons per unit surface area, per unit relative spectral interval). This can be obtained by summing up the electric field contribution from individual electrons within the beam and squaring, 

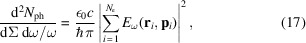

where *N*_ph_ is the number of photons, Σ is the surface area, *N*_e_ is the number of electrons in the beam, and **r**_*i*_ and **p**_*i*_ are the initial position and momentum of an electron, respectively. This sum can be decomposed into temporally coherent and incoherent parts (Hirschmugl *et al.*, 1991 ▸),

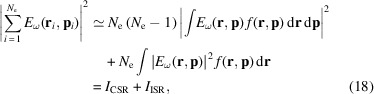

where *f*(**r**, **p**) is the electron beam distribution function. If the beam is long in comparison with the wavelength of the radiation, the coherent term can be neglected and the total intensity is then obtained by integrating the single electron intensity over *f*(**r**, **p**). Equation (18) contains six-dimensional integrals which are too numerically expensive to solve using standard quadrature methods. Therefore, a Monte Carlo approach is used instead. Here, the integrals are approximated as sums of the single electron electric field/intensity, calculated at the sample points {**r**_*i*_, **p**_*i*_} ≃ *f*(**r**, **p**). Fig. 3 ▸(*a*) shows an example of an intensity pattern for the system shown in Fig. 1 ▸, calculated using the method described here.

### 2.3. Wavefront propagation

The final part of the calculation involves propagating the wavefront through an optical system to the detector location. This is carried out using scalar diffraction theory (Goodman, 2005 ▸). Here, the paraxial approximation (small observation angles) is made, allowing us to treat the field components as independent and neglect the longitudinal field. Under this assumption, the propagation of a field through free space is given by the Rayleigh–Sommerfeld diffraction integral,

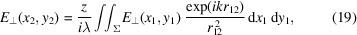

where *x*_1_, *y*_1_ and *x*_2_, *y*_2_ are transverse coordinates before and after propagation, respectively, *r*_12_ = [(*x*_2_ − *x*_1_)^2^ + (*y*_2_ − *y*_1_)^2^ + *z* ^2^]^1/2^ is the distance between positions on the two planes, Σ is the area of the source plane and *z* is the propagation distance. Equation (19) is a convolutional integral and can be written using the convolution theorem as 

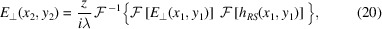

where 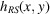 = 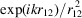 and 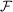 denotes a Fourier transform. This equation is solved numerically using a chirp *z* transform (CZT) implemented using Bluestein’s algorithm (Bluestein, 1970 ▸). A CZT is similar to the more common fast Fourier transform (FFT) but can be more computationally efficient. It allows the post-transform samples to be set independently of the input samples and internalizes zero padding of the wavefront (Leutenegger *et al.*, 2006 ▸).

At small observation angles, calculating 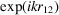 is prone to numerical errors. If this is the case we can make the Fresnel approximation, whereby a first-order Taylor expansion is used to give *r*_12_ ≃ *z* + 0.5(*x*_2_ − *x*_1_)^2^ + 0.5(*y*_2_ − *y*_1_)^2^. This is then inserted into equation (19) to give 

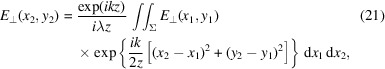

which is known as the Fresnel diffraction solution. This is also a convolutional type integral and is solved in the same way as equation (20).

To model the propagation through a lens or aperture the field is simply multiplied by transmittance functions. These transmittance functions are given by 

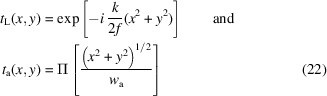

for a lens and aperture, respectively, where *f* is the lens focal length, Π(*x*) is the rectangle function and *w*_a_ is the aperture radius.

To measure the divergence of some incoming field, it can be imaged at the Fourier plane of a lens. We can model this system by multiplying the field by the lens transmittance function given by equation (22) and propagating *z* = *f* using equation (21). However, for low *f*/*#* lenses this calculation is prone to aliasing errors. Multiplying by the transmittance function introduces a quadratic phase term which has the effect of increasing the source bandwidth. A high density of samples in the initial wavefront is then required to avoid aliasing in the Fourier domain. This increases the memory requirement to propagate the field. To avoid this, we can directly substitute *E*(*x*_1_, *y*_1_) = *t*_L_(*x*_1_, *y*_1_)*E*(*x*_1_, *y*_1_) into equation (21) and rearrange to give 

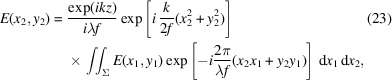

with the quadratic terms cancelling. This is known as the Fraunhofer diffraction integral and, unlike equations (19) and (21), it is not a convolution-type integral but simply a scaled Fourier transform.

Fig. 3 ▸(*b*) shows an example of a propagation calculation applied to the wavefront in Fig. 3 ▸(*a*). The field has been propagated to the Fourier plane of an *f* = 10 cm lens with a 2 cm-radius aperture. This calculation was performed using the Fraunhofer diffraction integral given by equation (23).

## 3. Benchmark simulations

To benchmark our implementation discussed in Section 2, we can compare simulations with analytical calculations of the emitted radiation. There are few systems in which this is possible, one being the emission from an electron performing circular motion in a constant magnetic field. The full calculation has been given by Jackson (1999 ▸) with the photon flux density (per unit solid angle dΣ = *R* ^2^dΩ) given as 

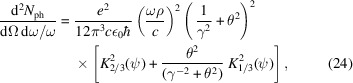

where ρ is the radius of curvature, θ is the polar angle, *K*_2/3_ and *K*_1/3_ are modified Bessel functions of the second kind and their argument is 

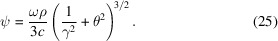

**Figure 4:**
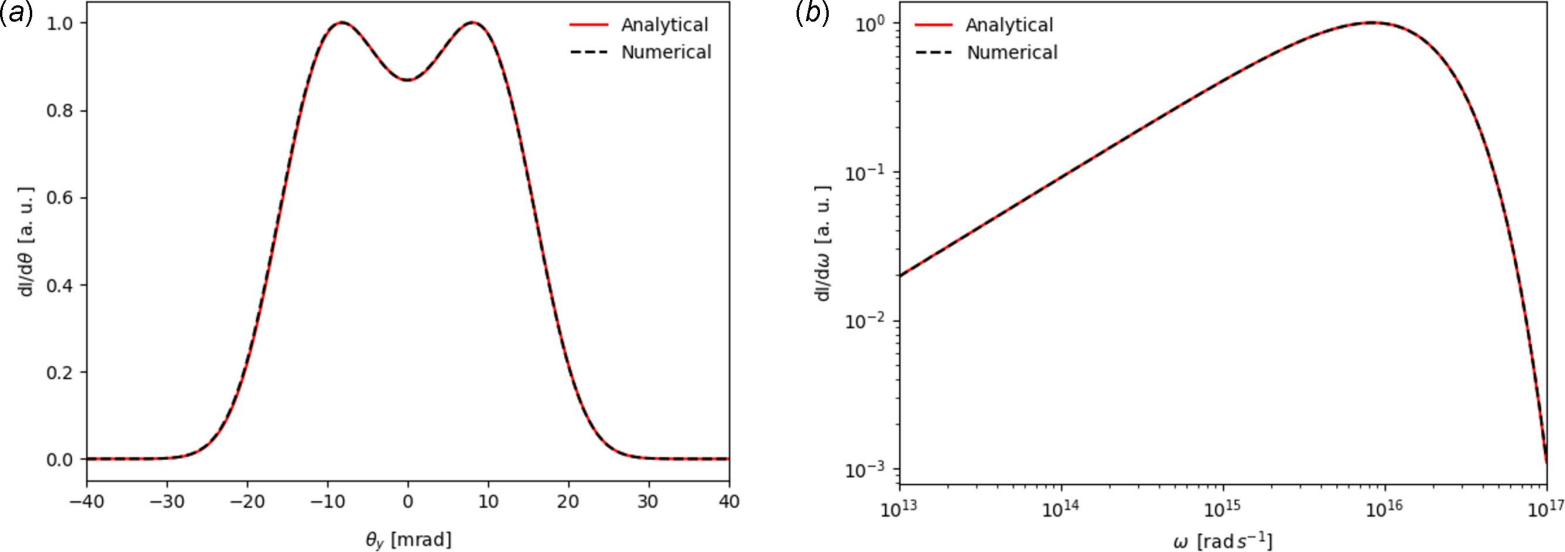
Circular motion photon flux density distribution calculated using both analytical (solid red line) and numerical (dashed black line) methods. Panel (*a*) shows the distribution against observation angle with fixed frequency ω = 10^16^ rad s^−1^. Panel (*b*) shows the distribution against frequency with an on-axis observation point (θ = 0).

Plots of equation (24) for fixed frequency and observation angle can be found in Figs. 4 ▸(*a*) and 4 ▸(*b*), respectively. These show the photon flux density emitted from a 100 MeV electron beam performing circular motion in a 1 T magnetic field. Also shown is the photon flux density calculated using the method discussed in Section 2. A very good agreement is seen between the two calculations.

**Figure 5:**
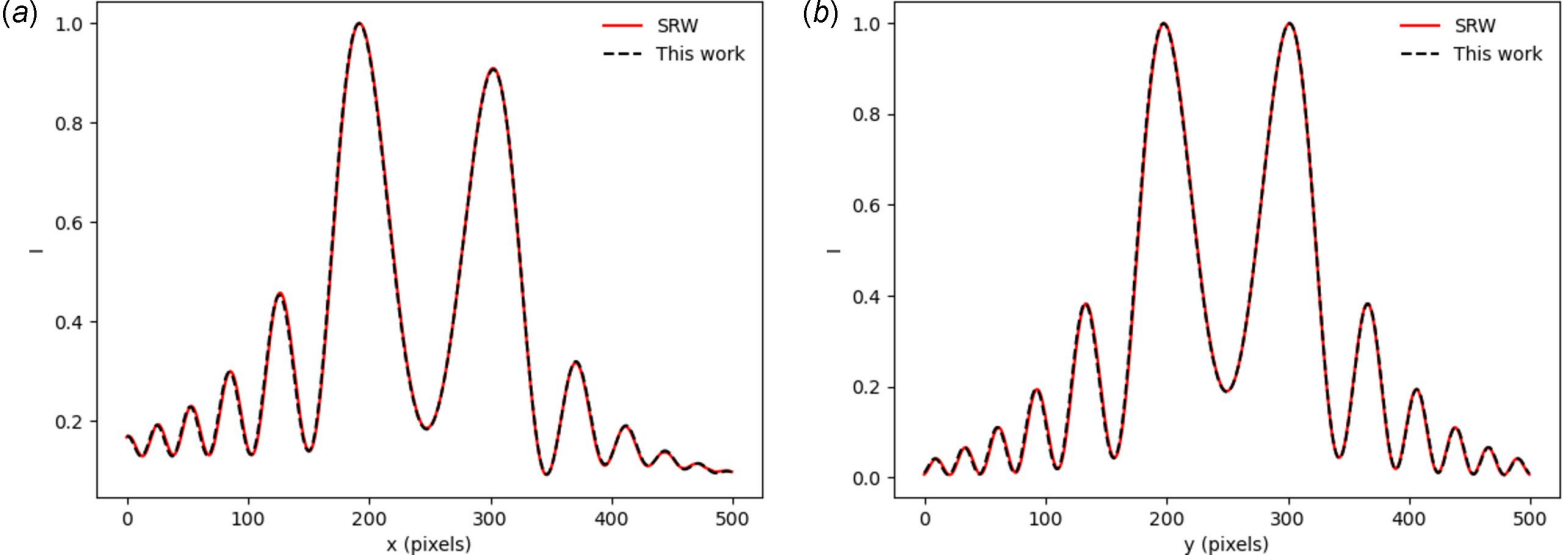
Photon flux density from a single electron calculated using *SRW* (solid red line) and *SYRIPY* (black dashed line). Panel (*a*) shows a horizontal lineout through the centre of the wavefront. Panel (*b*) shows a vertical lineout through the centre of the wavefront.

For the systems of interest to this work (*e.g.* Fig. 1 ▸) the emitted radiation distribution cannot be calculated analytically. Therefore, to benchmark this type of simulation we have compared results from *SYRIPY* with the widely used *SRW* package. This comparison can be found in Fig. 5 ▸, showing both horizontal and vertical lineouts of the flux density for a single electron, using the same simulation parameters as Fig. 3 ▸. Once again, the *SYRIPY* simulations agree well with the benchmark calculations.

When carrying out highly parallelized tasks, GPUs demonstrate superior performance compared with CPUs. As a result, optimizing *SYRIPY* for GPU execution offers a substantial performance advantage over *SRW*. A comparison of the performance of *SRW* and *SYRIPY* is shown in Table 1 ▸. Here, we can see the time taken to perform the benchmark calculation normalized by the number of macro electrons. The *SRW* simulations were performed using an AMD Ryzen Threadripper Pro 3955WX, 16-core, 32-Thread CPU, which was parallelized over multiple cores using Python’s multiprocessing package. The *SYRIPY* simulations were carried out using an NVIDIA A6000 RTX GPU in both single and double precision mode. This allows us to compare the performance of these two modes; however, for these simulation parameters the single precision error of ∼0.6% per pixel is acceptably low. In double precision mode, we find a modest improvement over *SRW*, with a ∼2× increase in the calculation rate. However, in single precision mode, a larger improvement of ∼15× is found.

In the above *SYRIPY* simulations, individual macro electrons were calculated sequentially. For large 2D wavefronts it is not possible to simulate multiple macro electrons in parallel, due to memory constraints. However, this is not the case for wavefronts with a lower number of samples (*e.g.* 1D simulations with *N*_*y*_ = 1). By calculating the individual macro electron wavefronts in batches, the overhead time is reduced, making more efficient use of the GPU resources. Table 1 ▸ also compares the performance for simulations with a 1D wavefront [in the horizontal plane, equivalent to Fig. 5 ▸(*a*)]. These simulations consisted of 10^6^ macro electrons, with a batch size of 10^4^ used for *SYRIPY*. In this case, *SYRIPY* shows a significant increase in calculation rate compared with *SRW* with an improvement of ∼9× for double precision mode and ∼53× for single precision mode.

## 4. Gradient-based Bayesian inference

The process of deducing latent variables from experimental measurements, such as inferring beam parameters from an observed intensity profile, is an example of an inverse problem that can be addressed using statistical inference techniques. The solution requires the implementation of a forward model of the system — typically a simulation. By adjusting the input parameters of the simulation, the output can be manipulated until it aligns with the experimental observations. *SYRIPY* has been designed primarily as a forward model for performing statistical inference of synchrotron-radiation-based diagnostics.

Solving inverse problems can be exceedingly computationally expensive if the number of latent variables is large. This is due to the high-dimensional space that must be searched, and is a direct consequence of the curse of dimensionality. Directly solving the equations in Section 2 using a C++/CUDA implementation would improve performance by removing the Python overhead. However, we elected to use *PyTorch* instead of CUDA as it allows us to make use of its automatic differentiation package. Gradient information allows us to search the input space in an intelligent manner, greatly reducing the resources required. This opens up the possibility of applying inference schemes which utilize a high-dimensional input space.

**Figure 6:**
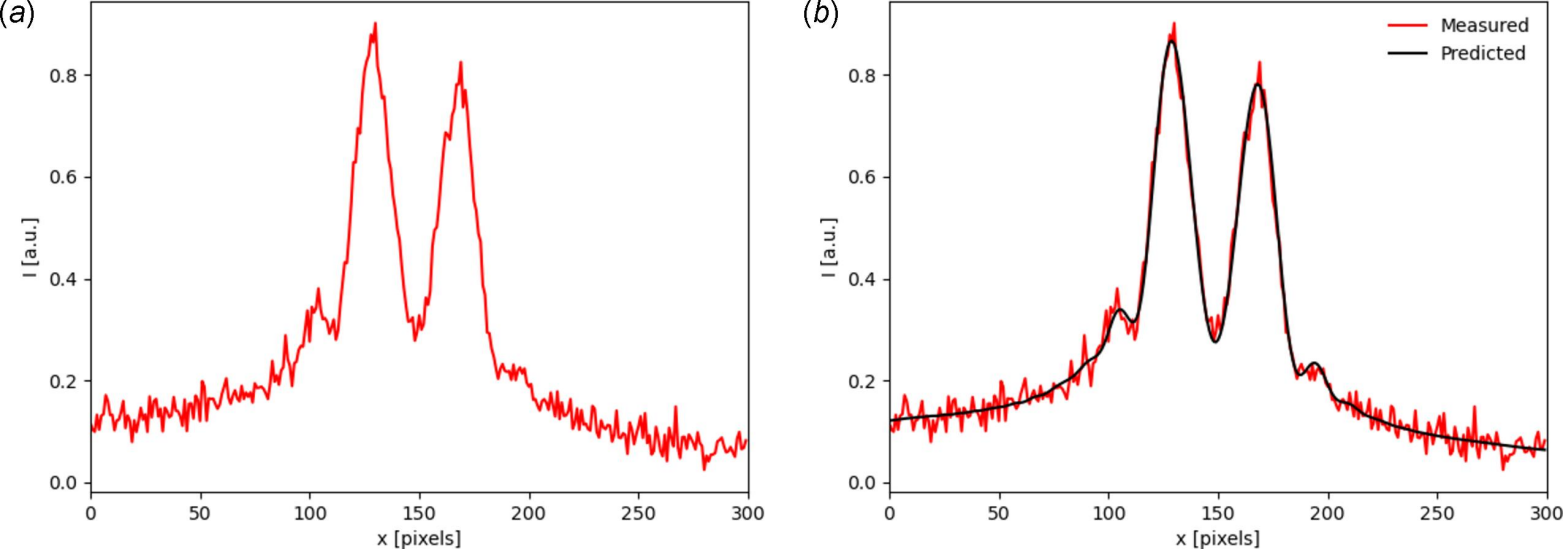
(*a*) Synthetic experiment data used to infer beam parameters with Bayesian inference. (*b*) Intensity profile using most probable input beam parameters.

Inverse problems are ill-posed when the forward model is not an injective function, *i.e.* multiple distinct inputs have the same output (Tarantola, 2005 ▸). For example, both the divergence and spot size of an electron beam contribute to a broadening of the emitted radiation. In this case, making a point estimate of the latent parameters may result in an erroneous result. Bayesian inference offers an approach for solving ill-posed inverse problems, as a probability distribution over the input spaces is inferred as opposed to a point estimate. On top of this, Bayesian methods offer a robust approach for uncertainty quantification. In this section, we will demonstrate how *SYRIPY* can be used to extract a distribution over beam size and divergence from a measured intensity profile. We will use the same setup as displayed in Fig. 1 ▸, but we omit the lens, making the intensity profile sensitive to the beam size. For this simplified example, we will restrict the measured intensity profile to 1D by taking a horizontal lineout through the centre of the wavefront. This reduces the number of parameters to infer, as the intensity profile only depends on the *x*-components of the beam size, σ_*x*_, and divergence, 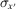 . The intensity profile used in this example is shown in Fig. 6 ▸(*a*), where we have assumed σ_*x*_ = 300 µm, 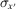 = 150 µrad and a Gaussian pixel noise with σ_*N*_ = 2% of the pixel counts.

We wish to obtain a joint probability distribution over all unknown parameters **x** = 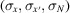 given the measured intensity profile **y**. This is achieved by applying Bayes’ rule 

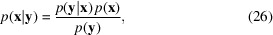

where *p*(**y**|**x**) is the likelihood, *p*(**x**) is the prior distribution and *p*(**y**) = 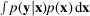 is the marginal distribution. The likelihood is the probability of obtaining the measured intensity profile given the input beam parameters. The pixel noise is Gaussian, so the appropriate form for the likelihood is 

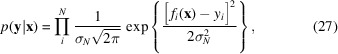

where *f*_*i*_(**x**) is the intensity obtained from the forward model at pixel *i*, *y*_*i*_ is the measured intensity at pixel *i* and *N* is the total number of pixels. The prior distribution encodes our prior knowledge of the beam parameters before observing **y**. We will only assume an order of magnitude knowledge of the parameters and use the following uninformative uniform priors: *p*(σ_*x*_) = 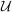 (100 µm, 1000 µm), 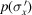 = 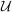 (100 µrad, 1000 µrad) and *p*(σ_*N*_) = 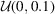 .

With *p*(**y**|**x**) and *p*(**x**) defined, equation (26) can be solved. However, carrying this out directly involves calculating *p*(**y**), an integral over all the beam parameters. With our simplified 1D example this is computationally expensive. However, for a more realistic higher-dimensional problem (*e.g.* including the *y*-dimension and finite energy spread), the integral is intractable. Therefore, an approximate inference scheme is required. Here, we will apply stochastic variational inference (SVI) (Hoffman & Blei, 2015 ▸), which uses a stochastic optimizer to solve equation (26) using the variational inference approximation. This involves assuming that the posterior can be approximated by a distribution with a known parametric form, *i.e.* *p*(**x**|**y**) ≃ *q*_**θ**_(**x**). This is known as the variational distribution with parameters **θ**. The objective of variational inference is to find the values of **θ** such that *q*_**θ**_(**x**) best approximates the true posterior. This is achieved by minimizing the distance between the two distributions. To do so requires a metric, such as the Kullback-Leibler (KL) divergence, 

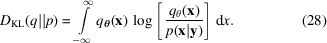

However, it is not possible to directly calculate *D*_KL_(*q*||*p*) as it requires prior knowledge of *p*(**x**|**y**) (the exact distribution we are trying to obtain). Therefore, to proceed, we can rewrite the KL divergence as 

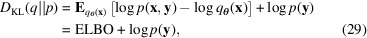

where 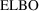 = 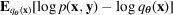 stands for the evidence lower bound (Hoffman & Blei, 2015 ▸). The second term in equation (29) is independent of **θ**; therefore, minimizing the KL divergence with respect to **θ** is equivalent to minimizing the ELBO, which can be directly calculated.

**Figure 7:**
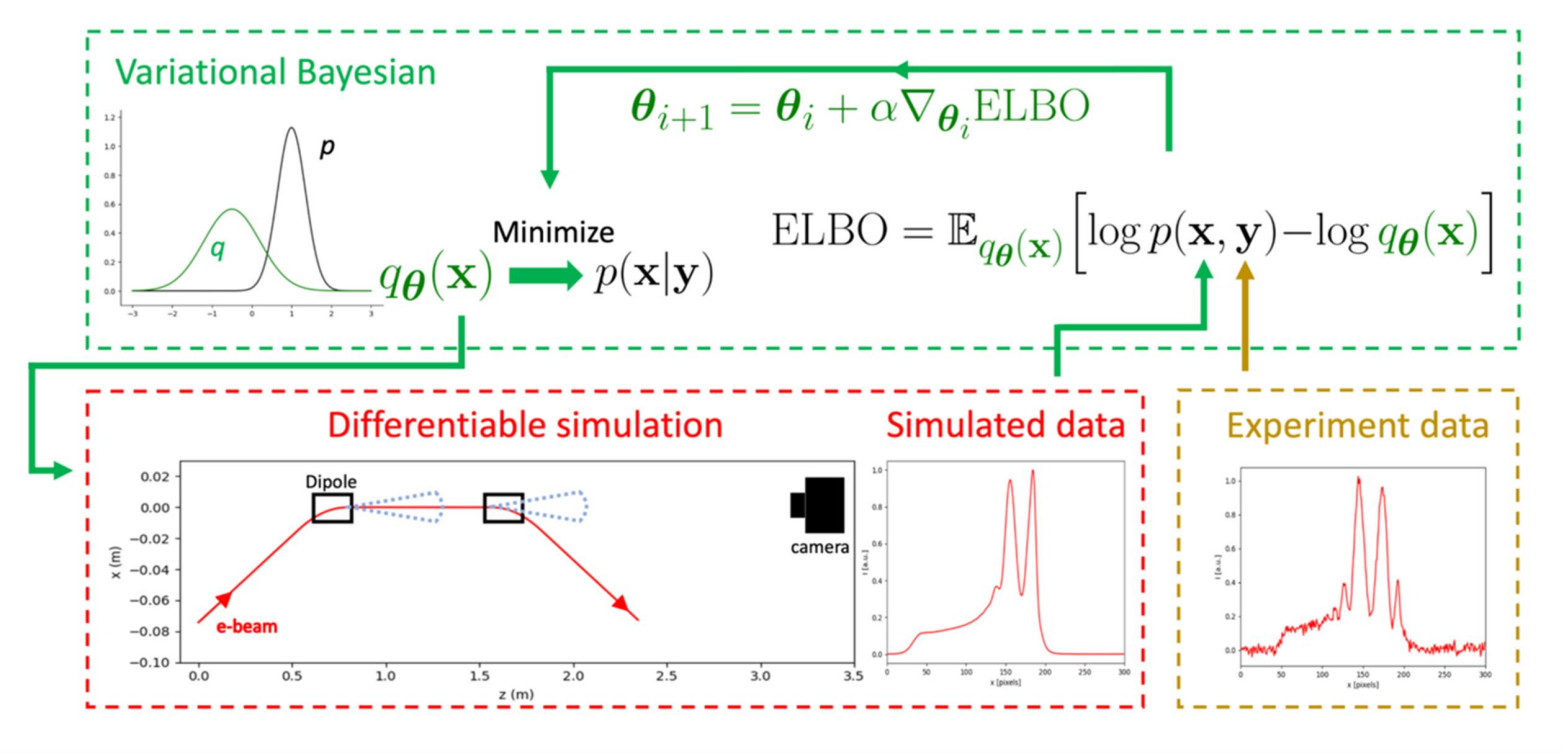
Flow diagram showing a variational Bayesian inference scheme using a differentiable simulation. The posterior distribution is approximated by a multivariate Gaussian *q*_**θ**_(**x**) with parameters **θ**. Samples are taken from this distribution and used as the inputs for a simulation. The simulation output and measured intensity profiles are used to calculate the ELBO where *p*(**x**, **y**) = *p*(**y**|**x**)*p*(**x**). Using automatic differentiation, the gradient of the ELBO with respect to **θ** is calculated. By minimizing the ELBO, the difference between *q*_**θ**_ and *p*(**x**|**y**) is also minimized.

**Figure 8:**
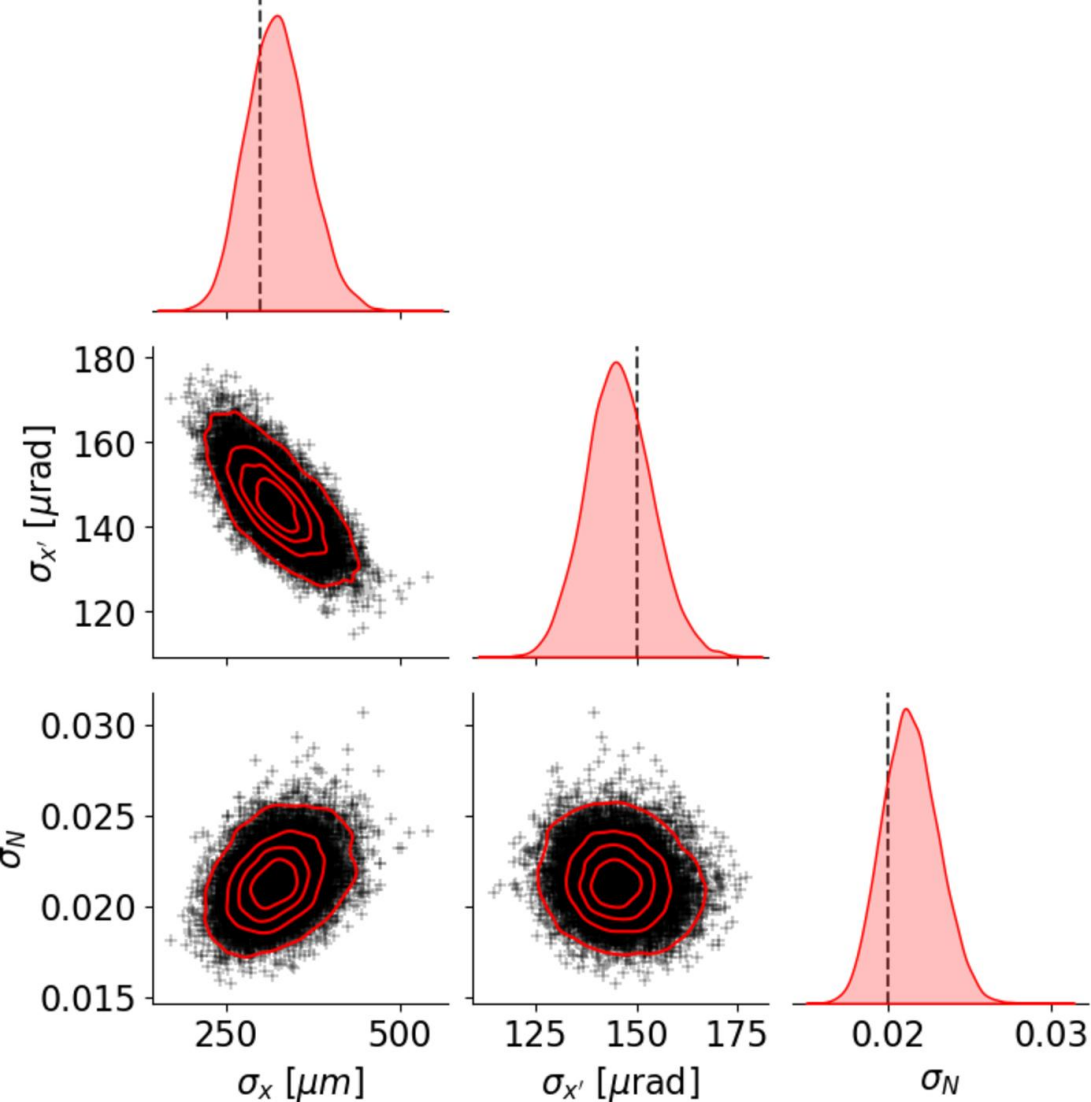
Pair plot of the posterior distribution showing all 2D and 1D marginals. The dashed vertical black lines correspond to the ground truth value.

Through this process the inference scheme has been reduced from an integral to an optimization problem, making it numerically tractable. During each update step ∇_**θ**_ELBO is required, which can be obtained efficiently as *SYRIPY* is differentiable. A flow diagram of the inference scheme is displayed in Fig. 7 ▸. To streamline the implementation of this scheme, the probabilistic programming library *Pyro* (Bingham *et al.*, 2018 ▸) has been used. A multivariate Gaussian was selected as the variational distribution, allowing for correlations between parameters to be accounted for. This results in a nine-dimensional optimization problem as we are inferring three means and six unique elements of the covariance. The result of applying SVI is displayed in Fig. 8 ▸, showing all the 2D and 1D marginals. A strong correlation between the beam size and divergence is shown, as expected. From the marginal distributions, we can extract estimates and uncertainties for the latent parameters giving σ_*x*_ = 323.6 ± 43.4, 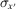 = 145.5 ± 8.1 and σ_*N*_ = 0.0213 ± 0.0017. The ground truth for each parameter lies within the 1σ bound for each estimate, and is shown in Fig. 8 ▸ with vertical dashed lines. Finally, the result of performing a forward simulation using the mode of the posterior (equivalent to the maximum *a posteriori* estimation) is shown in Fig. 6 ▸(*b*). A very good agreement between the synthetic experiment data and the most probable prediction is shown.

## 5. Summary

In this manuscript, we have introduced a novel Python package *SYRIPY*, specifically designed to facilitate the statistical inference of synchrotron-radiation-based diagnostics. *SYRIPY* is composed of three core modules: a particle tracker, a Liénard–Wiechert solver and a propagation module based on Fourier optics. This enables start-to-end simulations of the generation and detection of synchrotron radiation. The package has been developed using the library *PyTorch*, allowing *SYRIPY* to run natively on both CPUs and GPUs. In particular, the Liénard–Wiechert solver and the Fourier optics module are highly parallelizable, making the code highly efficient when run on a GPU. Developing the package with *PyTorch* as the underlying library enables the automatic calculation of gradients.

We have presented benchmark calculations, showing that the package agrees well with both analytical and numerical results. For simulations which only require single floating-point precision, *SYRIPY* shows a significant (∼50×) speed improvement compared with *SRW*. This is a direct result of the higher instruction throughput of a GPU when compared with a CPU.

*SYRIPY* is both fast and differentiable, making it an ideal tool for performing statistical inference. We have demonstrated this capability by using the package to perform Bayesian inference of simulated experimental data. With the application of SVI, the complex task of solving Bayes’ equation is reduced to a more manageable optimization problem. Even so, with our simplified 1D example, nine parameters must be optimized. This would be intractable without gradient information due to the curse of dimensionality. Moreover, the utility of *SYRIPY* is not limited to Bayesian inference. Other applications of the package could include inferring beam parameters rapidly through maximum likelihood estimation or predicting the full transverse phase space of the beam using a neural network parameterization as discussed by Roussel *et al.* (2023 ▸).

*SYRIPY* is publicly available on GitHub at https://github.com/robbiewatt1/SYRIPY.

## Figures and Tables

**Table 1:** Comparison between *SRW* and *SYRIPY* simulation run-time normalized by the number of macro electrons simulated

Wavefront shape	SRW Single-processor (s)	SRW Multiprocessor (s)	FP64 (s)	FP32 (s)
500 × 500 (2D)	1.86	0.086	0.039	0.0057
500 × 1 (1D)	0.014	0.00064	7.0 × 10^−5^	1.2 × 10^−5^
